# Ultrapulse Carbon Dioxide Laser in the Treatment of Secondary Scar Lesions of Acne Conglobata: A Novel, Minimally Invasive Method

**DOI:** 10.1111/jocd.71144

**Published:** 2026-08-20

**Authors:** Yuanyuan Xu, Mingzi Yang, Yuchen Zhang, Lianzhao Wang

**Affiliations:** ^1^ Department of Cicatrix Minimally Invasive Treatment Center, Plastic Surgery Hospital Chinese Academy of Medical Sciences and Peking Union Medical College Beijing China

**Keywords:** acne conglobata, laser therapy, scar

## Abstract

**Background:**

Acne conglobata (AC) is a severe, chronic relapsing inflammatory acne that often leads to disfiguring residual lesions, including sinus tracts, chronic abscesses, and epithelialized “skin bridges” on the face. Traditional surgical interventions for these sequelae have drawbacks such as prolonged recovery, risk of recurrence, and new scar formation, highlighting the need for minimally invasive effective therapies.

**Aims:**

This study aimed to evaluate the efficacy, safety, and patient tolerance of ultrapulse carbon dioxide (CO_2_ ) laser therapy for scar lesions secondary to AC.

**Methods:**

Patients diagnosed with AC who received ultrapulse fractional CO_2_ laser therapy between January 2022 and January 2026 were retrospectively analyzed. Treatment outcomes were evaluated by recurrence rate, patient satisfaction, Global Improvement Score, wound healing time, and complications.

**Results:**

A total of 13 patients were enrolled in this study. A total of 301 skin bridges, 51 sinus tracts, and 8 chronic abscesses were treated. The median wound healing time was 22 (20, 23.5) days, and no complications were observed. No lesion recurrence was noted during the follow‐up period (median 11 months). All patients were satisfied with their posttreatment outcomes.

**Conclusions:**

Ultrapulse CO_2_ laser therapy may represent a feasible minimally invasive option for chronic structural lesions secondary to AC, with favorable clinical outcomes and acceptable tolerability.

## 1 Introduction

Acne conglobata (AC) is a severe, recurrent form of inflammatory acne and a core component of the follicular occlusion tetrad, a spectrum of chronic disorders that share the pathogenic features of follicular occlusion and perifolliculitis [1]. It predominantly affects young adults, with characteristic lesions frequently occurring on the face, which is a site of significant cosmetic and psychosocial concern. The clinical presentation on the face is characterized by the development of deep, tender, and often interconnected inflammatory nodules and abscesses. As the disease follows a chronic relapsing course, it frequently leads to extensive and disfiguring scarring. These residual lesions are not merely changes to the skin texture but often include persistent sinus tracts, chronic fluctuating abscesses, and distinctive epithelialized “skin bridges.” The presence of these destructive and deforming lesions in highly visible areas causes pain and infection, imposes a profound psychological burden on patients, and severely impairs their quality of life [2].

The management of AC and residual scars is challenging. Systemic therapies including isotretinoin, antibiotics, and biologics are the main strategies for controlling active inflammation [3, 4]. Surgical interventions have been considered for established scar lesions and sinus tracts. Traditional surgical options include incision, drainage, deroofing, and wide local excision [5]. However, these procedures are often associated with drawbacks such as intraoperative bleeding, risk of infection, prolonged wound healing, and the potential for recurrence or new scar formation [4, 5, 6]. Moreover, for extensive lesions, primary closure may be impossible, necessitating complex reconstructive procedures, such as skin grafting or flap plasty, which are less tolerated by patients, especially in the facial region [4, 5, 6].

Given these limitations, there is a need for minimally invasive, yet effective modalities to address the structural sequelae of AC. In this study, we propose a novel therapeutic approach utilizing an ultrapulse carbon dioxide (CO_2_) laser to treat scar lesions secondary to AC. With the aim to employ the ablative and thermal effects of the laser to open the sinus tracts precisely, remove epithelialized skin bridges, and alleviate epidermal folding at the lesion site. Fractional photothermolysis promotes dermal remodeling, repairs damaged skin structures, and modulates local inflammatory responses. Compared to traditional extensive surgery, this method aims to control recurrent subclinical infections and improve scar appearance without requiring complete lesion resection, thereby offering the potential benefits of minimal damage, rapid recovery, and fewer complications, which are particularly crucial for facial treatment.

## 2 Methods and Materials

### 2.1 Patients

Thirteen patients diagnosed with AC who underwent ultrapulse fractional CO_2_ laser therapy between January 2022 and January 2026 were included in this study. The treatment target was not active inflammatory AC itself, but chronic structural sequelae caused by previous inflammation, including epithelialized skin bridges, sinus tracts, and chronic cavities. Patients with uncontrolled acute inflammatory exacerbation, rapidly progressive infection, or lesions requiring urgent drainage were excluded. Medical records, including sex, age, disease duration, and number of CO_2_ laser‐treated lesions, were retrospectively reviewed. Pre‐ and postoperative photographs were obtained for all patients. All procedures were performed in accordance with the ethical standards of the institutional committee and the 1964 Declaration of Helsinki along with its later amendments or comparable ethical standards. Informed consent was obtained from all the participants.

### 2.2 Treatment Technique

**FIGURE 1:**
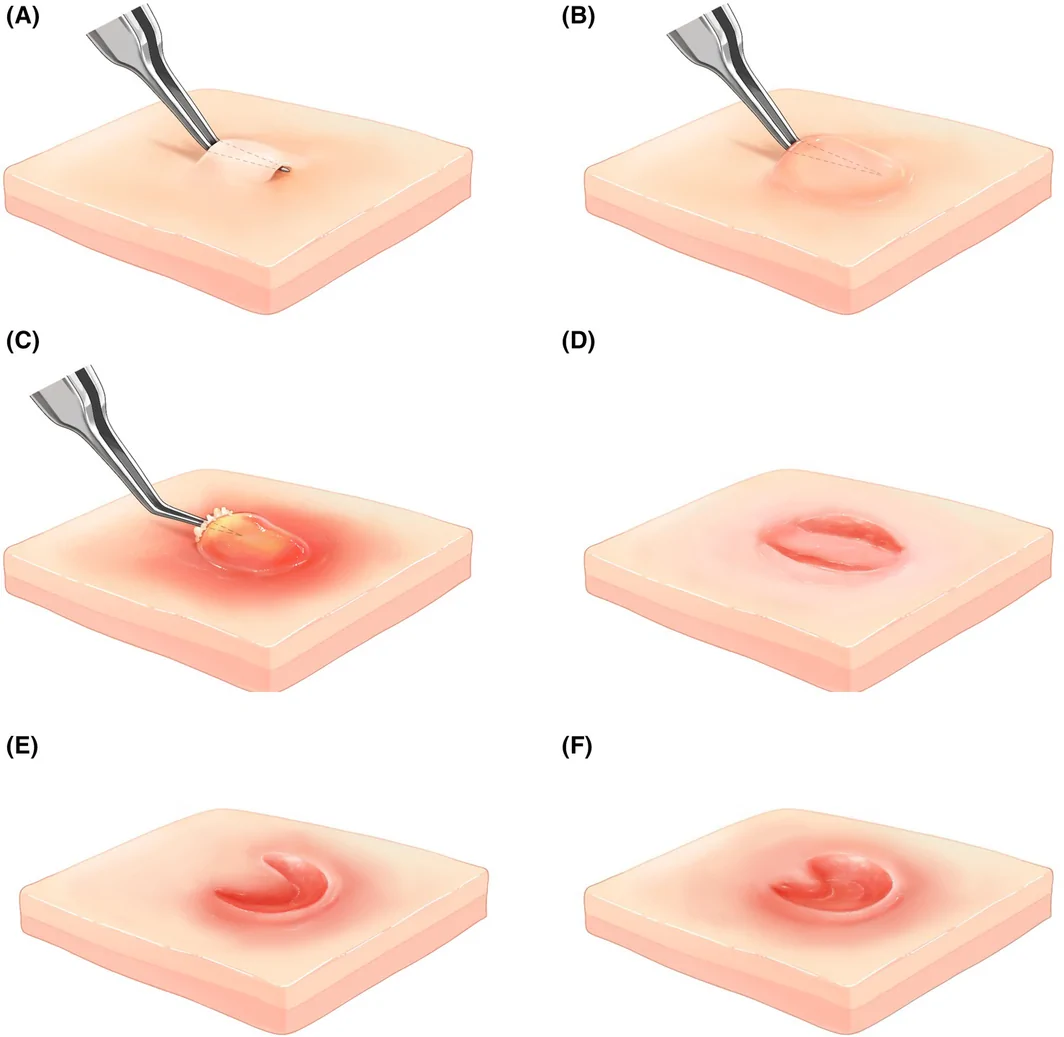
Schematic of laser treatment. (A–C) The use of blunt‐tipped tweezers to explore the skin bridge, sinus tract, and chronic abscess, respectively. (D) Appearance after laser treatment of the skin bridge lesion with the overlapping epidermis removed and the underlying normal epidermis exposed. (E) The appearance after laser treatment of the sinus tract lesion, with the surface epidermal roof removed and the normal epidermis below exposed. (F) Appearance after laser treatment of a chronic abscess lesion with the cavity open.

All procedures were performed in outpatient facilities under local anesthesia. Local infiltration anesthesia with 0.5% lidocaine, 0.25% ropivacaine, and 0.01% epinephrine was administered. Lesions were explored using blunt‐tipped tweezers. An ultrapulse CO_2_ laser (10 600 nm) was used to treat skin lesions at a spot size of 0.2 mm, 15 mJ, and 1 W. For sinus tracts and abscesses, the lesions were opened under the guidance of blunt‐tipped tweezers, and the entire roof was vaporized. Skin bridges were directly transected and removed. The principle of CO_2_ laser treatment is to remove the overlapping epidermis rather than to completely resect the entire lesion down to the subcutaneous fat layer. A schematic of the laser treatment is shown in Figure 1. After the procedure, Longzhu ointment was applied to the wound. Patients were instructed to clean the wound twice daily with normal saline and then topically apply Longzhu ointment for the first 7 days. Thereafter, Longzhu ointment was applied daily after washing until the wound healed completely.

### 2.3 Evaluation of Treatment Results

The clinical outcomes of CO_2_ laser treatment were evaluated based on recurrence, patient‐reported satisfaction, and investigator‐assessed photographic improvement. Recurrence was defined as the formation of new abscesses, sinus tracts, or skin bridges within the treated scar site, or within 5 mm of the scar. Patient satisfaction was assessed using a self‐reported 5‐point satisfaction scale after treatment (1 = poor, 2 = fair, 3 = good, 4 = very good, and 5 = excellent). To provide an additional assessment of clinical improvement, standardized pre‐ and postoperative photographs were independently evaluated by two observers who were not involved in patient treatment. The overall clinical improvement was graded using a Global Improvement Score (GIS), ranging from 0 to 4 (0 = no improvement, 1 = mild improvement, 2 = moderate improvement, 3 = marked improvement, and 4 = almost complete resolution). Safety was evaluated based on the wound healing time and whether any complications occurred.

### 2.4 Statistical Analysis

Statistical analyses were performed using IBM SPSS Statistics for Windows (version 22.0; IBM Corp., Armonk, NY, USA). Given the lack of a normal distribution, continuous variables were expressed as medians (interquartile range).

## 3 Results

Thirteen patients with secondary scar lesions from AC were included in this study. The general clinical characteristics and lesion conditions are summarized in Table 1. The median age of the patients at the time of the first laser treatment was 28 (21, 30) years, and the median duration of AC was 7 (5, 10) years. In terms of sex distribution, there were 9 males (69%) and 4 females (31%). A total of 301 skin bridges, 51 sinus tracts, and eight chronic abscesses were treated. This distribution is consistent with the expected clinical manifestations of secondary scars from AC. At the time of laser treatment, none of the patients had uncontrolled acute inflammatory exacerbation or progressive infection requiring systemic escalation. Five patients had previously received isotretinoin treatment before laser therapy. However, none of the patients received isotretinoin, antibiotics, or biologic agents concurrently during laser treatment or throughout follow‐up.

**TABLE 1:** Characteristics of the 13 patients.

Characteristic	Value
Age at first laser treatment, median (interquartile range), years	28 (21, 30)
Disease duration, median (interquartile range), years	7 (5, 10)
Sex, Number (%)	
Female	4 (31%)
Male	9 (69%)
Number of treated lesions	
Skin bridges	301
Sinus tracts	51
Chronic abscesses	8

**FIGURE 2:**
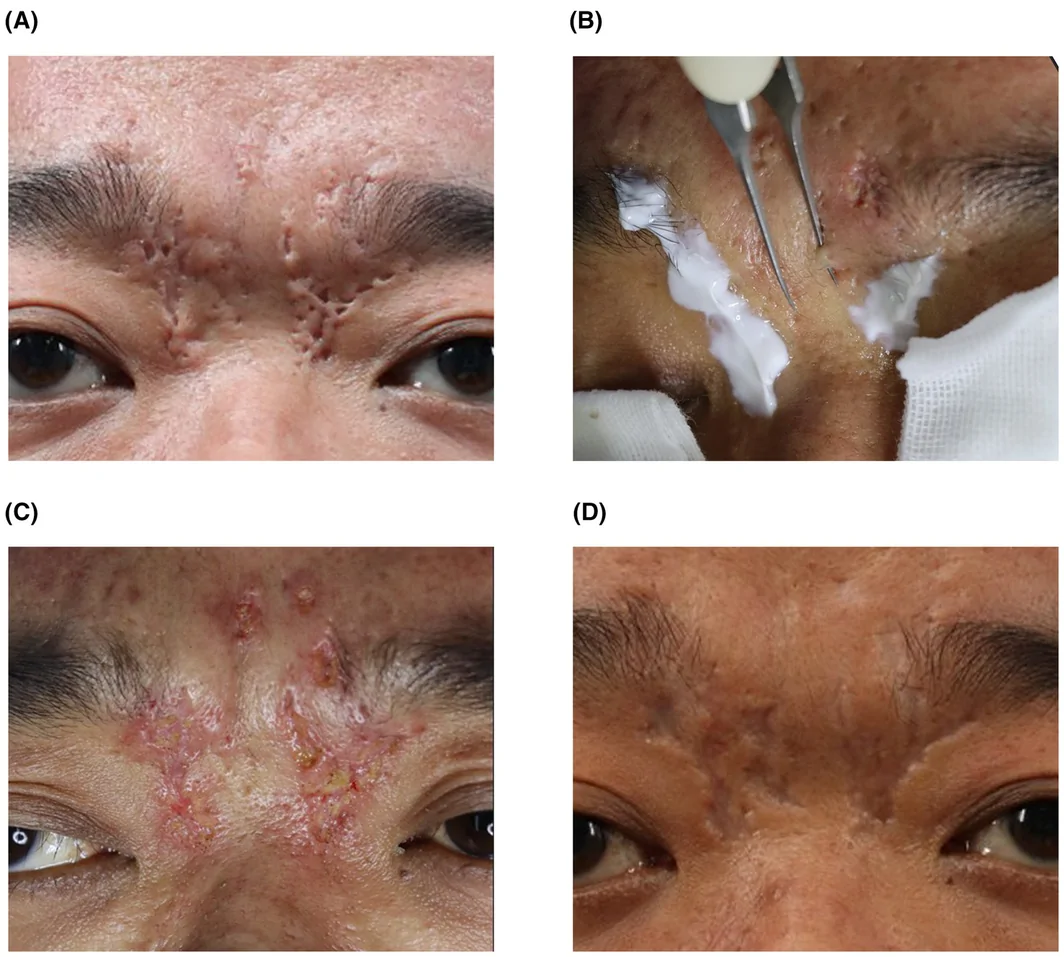
(A) Preoperative views of a 26‐year‐old male with AC who underwent ultrapulse CO_2_ laser treatment. (B) During treatment, use the blunt‐tipped tweezer to explore lesions. (C) Appearance immediately after treatment. The sinus tracts were opened, and the skin bridges were removed. (D) Postoperative views 11 months after treatment. The skin bridges and sinus tracts have been completely removed, and no recurrence has been observed.

**FIGURE 3:**
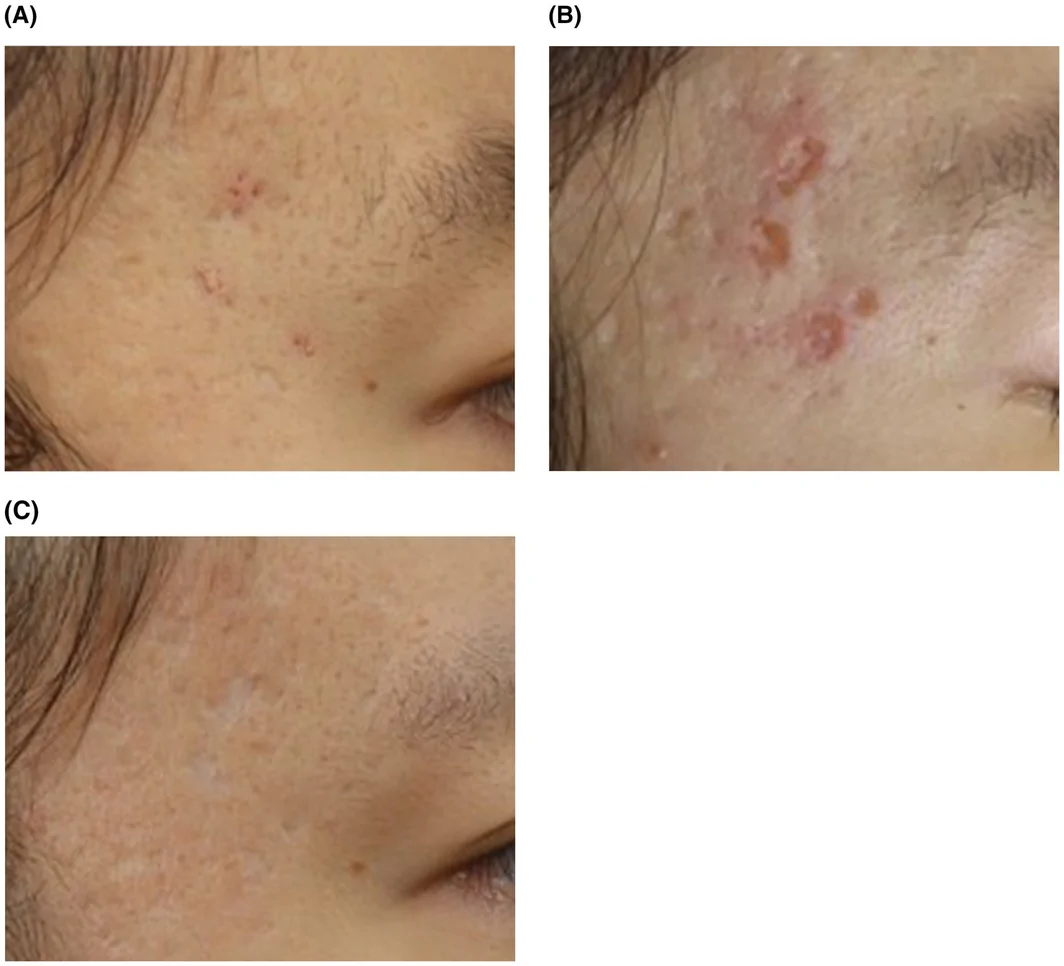
(A) Preoperative views of a 21‐year‐old female with AC who underwent ultrapulse CO_2_ laser treatment. (B) Appearance immediately after treatment. (C) Postoperative views 13 months after treatment. The skin bridges and sinus tracts have been completely removed, and no recurrence has been observed. The appearance of this patient has significantly improved.

All laser treatments for the 13 enrolled patients were performed on an outpatient basis under topical or infiltration anesthesia. The median visual analog scale score for pain during laser treatment reported by the patients was 4 (3, 5), indicating mild and controllable pain. The median wound healing time after treatment was 22 (20, 23.5). No relevant complications, such as bleeding, infection, hyperpigmentation, or scar hyperplasia, were observed. All patients completed the follow‐up, with a median follow‐up duration of 11 (2.5, 23) months. Individual follow‐up durations and recurrence status are summarized in Table S1. No recurrence of treated structural lesions was observed during the follow‐up period. All patients rated their satisfaction with the treatment outcome as 5 points (excellent). In the blinded photographic assessment, the median GIS score was 3 (2.5, 3). Both independent observers observed clinical improvement after treatment, with most patients achieving marked improvement. These findings suggest that ultrapulse CO_2_ laser therapy for scar lesions secondary to AC is not only effective and safe, but also associated with good patient tolerance and satisfaction (Figures 2 and 3).

## 4 Discussion

This study employed an ultrapulse CO_2_ laser to treat secondary scar lesions resulting from AC, achieving favorable therapeutic outcomes. The primary role of this approach is local correction of chronic structural abnormalities rather than modification of the systemic inflammatory activity of AC. The ultrapulse treatment demonstrated significant effects in improving scar appearance and restoring skin smoothness. The treatment regimen was safe, with no serious adverse reactions occurring during or after the procedure, and patient tolerance was high. This indicates that the ultrapulse CO_2_ laser is an effective and safe tool for managing the chronic, refractory sequelae of AC.

For sinus tracts, chronic abscesses, and skin bridges secondary to AC, the traditional treatment methods primarily include incision and drainage, unroofing, and surgical excision. Although these methods can directly remove part of the lesional tissue, they have significant limitations, such as relatively high trauma; prolonged recovery periods; a tendency to form new, noticeable scars; and incomplete treatment of deep, interconnected lesions, leading to a high disease recurrence rate [3, 7, 8, 9]. By contrast, laser‐based treatment strategies offer unique advantages. The core therapeutic mechanism of the ultrapulse CO_2_ laser used in this study was the utilization of the laser's high energy and strong absorption by water to achieve precise vaporization and excision of pathological tissue. Compared to traditional surgical methods, laser treatment has several advantages: First, it is minimally invasive and precise; laser treatment allows for precise control of the depth and scope of action, causing minimal damage to surrounding normal tissue. Second, it combines sterilizing and anti‐inflammatory effects, as the high temperature of the laser can kill bacteria (such as *Cutibacterium acnes*) residing within hair follicles and sinus tracts, and seal microvessels, thereby reducing the local inflammatory response and lowering the risk of recurrence at its source. Finally, the laser treatment promotes the repair of tissue, the thermal stimulation from the laser promotes collagen remodeling and regeneration in the dermis, facilitating the repair of structural defects [10, 11, 12].

This study approached AC treatment with a different perspective than traditional treatment methods. AC is a severe manifestation of follicular occlusion syndrome. Its pathological basis is follicular hyperkeratosis, which leads to the occlusion and dilation of the pilosebaceous unit, subsequently rupturing and triggering a severe foreign‐body granulomatous inflammatory reaction. This ultimately becomes chronic and causes severe destruction of skin structure, forming scars, subcutaneous sinus tracts, and “skin bridges.” Traditional laser or surgical excision often aims for complete removal of the lesion, frequently requiring penetration into the subcutaneous fat layer, resulting in significant trauma [12, 13, 14, 15]. In contrast, our method targeted the structural abnormalities of scars, sinus tracts and skin bridges, utilizing the cutting and vaporizing functions of the ultrapulse CO_2_ laser to perform precise lesion debridement and structural reconstruction. Specifically, this involves using the laser to excise the skin bridges that cause persistent inflammation and poor drainage, open closed sinus tracts, relieve physical follicular occlusion, and create channels for the discharge of deep inflammatory material, allowing for subsequent tissue repair. This method addresses the relationship between disease chronicity and structural destruction, effectively controlling local inflammation and initiating the physiological repair process. Compared to traditional procedures such as incision and drainage, complete lesion excision, or skin grafting, this method does not require extensive removal of the lesion and large amounts of normal tissue; however, this minimally invasive treatment can eliminate active lesions and promote healing, while causing less damage, posing lower risks, and enabling faster recovery.

Our study had several limitations. First, it is important to clarify that this laser treatment regimen primarily targets scarring and structural lesions secondary to the chronic phase of AC but not acute infectious abscesses. Its primary purpose is to relieve structural occlusion, control chronic inflammation, and create conditions for tissue repair, not primarily to prevent infection. Therefore, for lesions with obvious active infections, a combination of pharmacological treatments is necessary. Second, in terms of aesthetic repair, this method mainly addresses lesion activity and gross structural abnormalities. For residual post‐treatment issues, such as uneven skin texture, fine lines, pigmentation, and deep depressions, a single treatment session is often insufficient. To achieve ideal aesthetic improvement, it is typically necessary to use this as a foundation and subsequently combine it sequentially with comprehensive treatment modalities, such as PLASMA, non‐ablative/ablative fractional lasers, or subcision and filling. Future research should focus on exploring the optimal combination models and timing of structural abnormality‐relieving laser therapy with various reparative treatments to develop individualized sequential treatment plans that maximize the restoration of patient function and appearance. Furthermore, although the median follow‐up duration was 11 months, longer follow‐up is required given the chronic relapsing nature of AC. In addition, although blinded photographic assessment using the GIS was incorporated, the retrospective design, limited sample size, and lack of standardized objective outcome measures and quality‐of‐life assessments remain limitations. Future prospective studies with larger sample sizes, longer follow‐up, and comprehensive outcome assessments are needed to further validate this approach.

## 5 Conclusions

In this retrospective case series, ultrapulse CO_2_ laser therapy demonstrated a promising minimally invasive approach for chronic structural lesions secondary to AC, including epithelialized skin bridges, sinus tracts, and chronic cavities. The treatment was associated with favorable clinical improvement, high patient satisfaction, and acceptable safety during follow‐up. Further prospective studies with larger sample sizes, longer follow‐up, and standardized outcome assessments are warranted to validate the long‐term efficacy and durability of this approach.

## Author Contributions

Yuanyuan Xu and Mingzi Yang designed this study and completed the article. Yuchen Zhang helped to collect the data for this study. Lianzhao Wang revised the article. Yuanyuan Xu and Mingzi Yang have contributed equally to this work and should be regarded as co‐first authors.

## Funding

This work was supported by the Key Medical Discipline Research Project of Beijing Shijingshan District and the Plastic Surgery Hospital, Chinese Academy of Medical Sciences (Grant No. YS2024CG001).

## Ethics Statement

All procedures were performed in accordance with the ethical standards of the institutional committee and the 1964 Declaration of Helsinki along with its later amendments or comparable ethical standards. Informed consent was obtained from all the participants.

## Conflicts of Interest

The authors declare no conflicts of interest.

## Supporting information


**Table S1:** Individual follow‐up duration and recurrence status of enrolled patients.

## Data Availability

The data that support the findings of this study are available from the corresponding author upon reasonable request.
