# Supplementary material for: Ultrapulse Carbon Dioxide Laser in the Treatment of Secondary Scar Lesions of Acne Conglobata: A Novel, Minimally Invasive Method
Source: J Cosmet Dermatol. 2026 Aug 20;25(8):e71144. doi: 10.1111/jocd.71144 (PMC13494063; doi:10.1111/jocd.71144)
Supplement: Supplementary file 1 [file JOCD-25-e71144-s001.docx]

**Supplementary Table 1. Individual follow-up duration and recurrence status of enrolled patients**

| Patient | Follow-up duration (months) | Recurrence of treated lesions |
| --- | --- | --- |
| Patient 1 | 1 | No |
| Patient 2 | 2 | No |
| Patient 3 | 2 | No |
| Patient 4 | 3 | No |
| Patient 5 | 8 | No |
| Patient 6 | 9 | No |
| Patient 7 | 11 | No |
| Patient 8 | 11 | No |
| Patient 9 | 13 | No |
| Patient 10 | 22 | No |
| Patient 11 | 24 | No |
| Patient 12 | 25 | No |
| Patient 13 | 26 | No |
